# Supplementary material for: Complex‐centric proteome profiling by SEC‐SWATH‐MS
Source: Mol Syst Biol. 2019 Jan 14;15(1):e8438. doi: 10.15252/msb.20188438 (PMC6346213; doi:10.15252/msb.20188438)
Supplement: Supplementary file 7 — Dataset EV6 [file MSB-15-e8438-s007.zip › feature_plots_bioplex/Q9UKZ9.pdf]

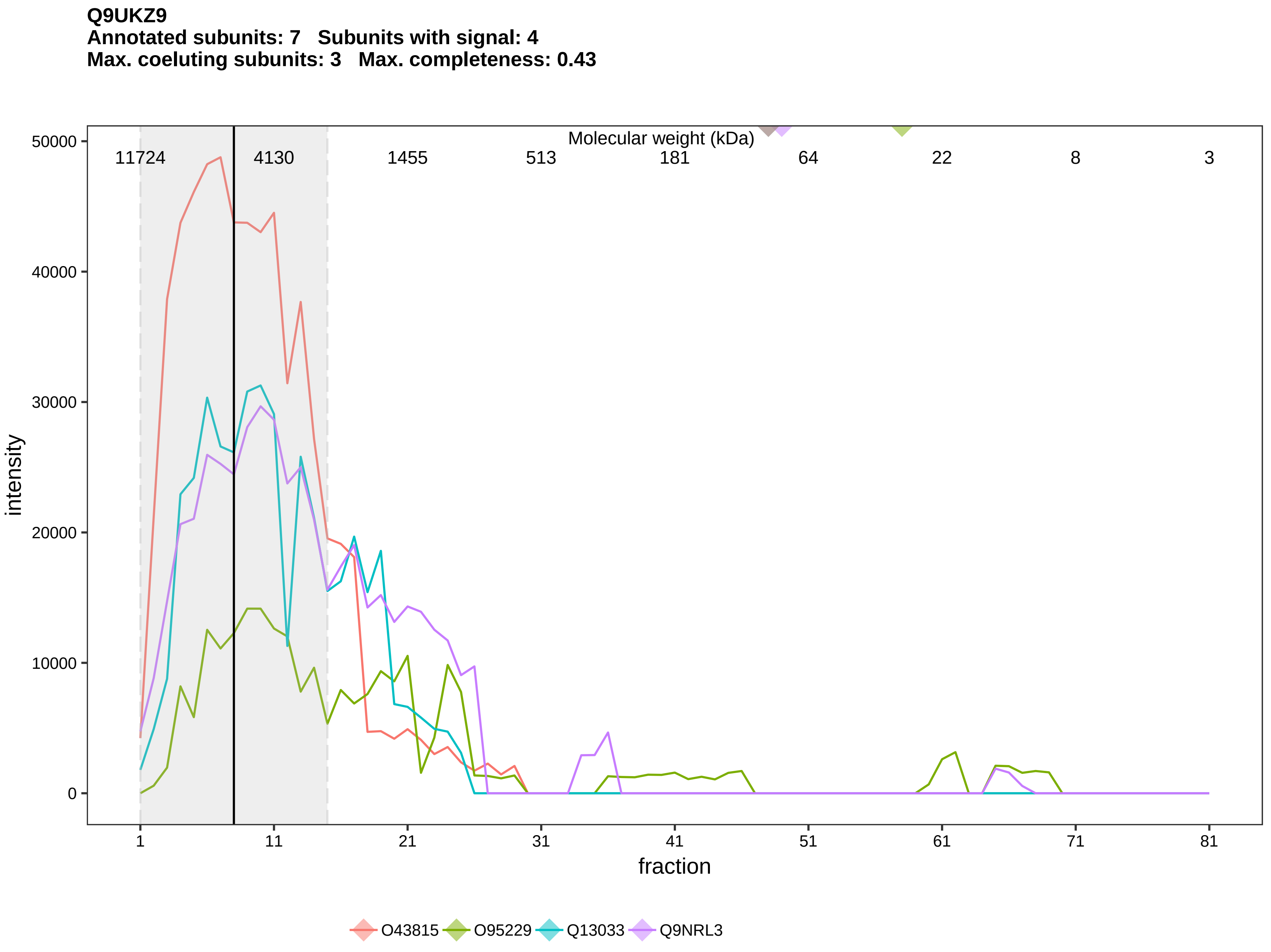

Q9UKZ9
Annotated subunits: 7 Subunits with signal: 4
Max. coeluting subunits: 3 Max. completeness: 0.43
Molecular weight (kDa)
11724
4130
1455
513
181
64
22
8
3
intensity
50000
40000
30000
20000
10000
0
1
11
21
31
41
51
61
71
81
fraction
O43815
O95229
Q13033
Q9NRL3
